# Identification of Potential Biomarkers From Hepatocellular Carcinoma With MT1 Deletion

**DOI:** 10.3389/pore.2021.597527

**Published:** 2021-04-01

**Authors:** Ruohao Zhang, Miao Huang, Hong Wang, Shengming Wu, Jiali Yao, Yingying Ge, Yufei Lu, Qiping Hu

**Affiliations:** ^1^ Department of Cell Biology and Genetics, School of Pre-clinical Medicine, Guangxi Medical University, Nanning, China; ^2^ Radiology Department, Affiliated Tumor Hospital of Guangxi Medical University, Nanning, China; ^3^ Department of Pathology, Affiliated Tumor Hospital of Guangxi Medical University, Nanning, China; ^4^ Department of Histology and Embryology, School of Pre-clinical Medicine, Guangxi Medical University, Nanning, China

**Keywords:** hepatocellular carcinoma, copy number variation, metallothionein, hub gene, deletion, screening, biomarker

## Abstract

**Background:** Hepatocellular carcinoma (HCC) is one of the deadliest cancers worldwide. Metallothioneins (MTs) are metal-binding proteins involved in multiple biological processes such as metal homeostasis and detoxification, as well as in oncogenesis. Copy number variation (CNV) plays a vital role in pathogenesis and carcinogenesis. Nevertheless, there is no study on the role of MT1 CNV in HCC.

**Methods:** Array-based Comparative Genomic Hybridization (aCGH) analysis was performed to obtain the CNV data of 79 Guangxi HCC patients. The prognostic effect of MT1-deletion was analyzed by univariate and multivariate Cox regression analysis. The differentially expressed genes (DEGs) were screened based on The Gene Expression Omnibus database (GEO) and the Liver Hepatocellular Carcinoma of The Cancer Genome Atlas (TCGA-LIHC). Then function and pathway enrichment analysis, protein-protein interaction (PPI) and hub gene selection were applied on the DEGs. Lastly, the hub genes were validated by immunohistochemistry, tissue expression and prognostic analysis.

**Results:** The MT1-deletion was demonstrated to affect the prognosis of HCC and can act as an independent prognostic factor. 147 common DEGs were screened. The most significant cluster of DEGs identified by Molecular Complex Detection (MCODE) indicated that the expression of four MT1s were down-regulated. MT1X and other five hub genes (TTK, BUB1, CYP3A4, NR1I2, CYP8B1) were associated with the prognosis of HCC. TTK, could affect the prognosis of HCC with MT1-deletion and non-deletion. NR1I2, CYP8B1, and BUB1 were associated with the prognosis of HCC with MT1-deletion.

**Conclusions:** In the current study, we demonstrated that MT1-deletion can be an independent prognostic factor in HCC. We identified TTK, BUB1, NR1I2, CYP8B1 by processing microarray data, for the first time revealed the underlying function of MT1 deletion in HCC, MT1-deletion may influence the gene expression in HCC, which may be the potential biomarkers for HCC with MT1 deletion.

## Introduction

Globally, liver cancer is one of the leading causes of cancer death. HCC, a predominant type of liver cancer, which counts for approximately 80% of all the liver cancer cases [1]. Nevertheless, the underlying molecular mechanisms of HCC is currently unclear.

CNV is caused by the rearrangement of the genome. It generally refers to the duplication or deletion of large fragments of the genome with a length of more than 1 kB, can activate oncogenes and neoplastic pathways [2]. Some studies have shown that copy number variation in HCC may affect gene expression alterations, thus affecting the occurrence and development of HCC [3, 4]. Genomic copy number deletion at chromosome 14q31.1-32.13 was frequently observed in HCC, as well as zinc finger CCCH-type containing 14 (ZC3H14) is located on 14q31.1-32.13, which was demonstrated to be related with HCC [5].

Metallothioneins (MTs) are metal-binding proteins involved in diverse processes, including metal homeostasis and detoxification, oxidative stress response, and cell proliferation, which have four main isoforms (MT1, MT2, MT3, and MT4) [6, 7]. Emerging studies have demonstrated that the abnormal expression of MTs, such as MT1 are able to trigger the process of carcinogenesis in various types of human malignancies, including HCC [8]. However, currently, there is no research on the relationship between MT1 CNV and HCC.

**FIGURE 1:**
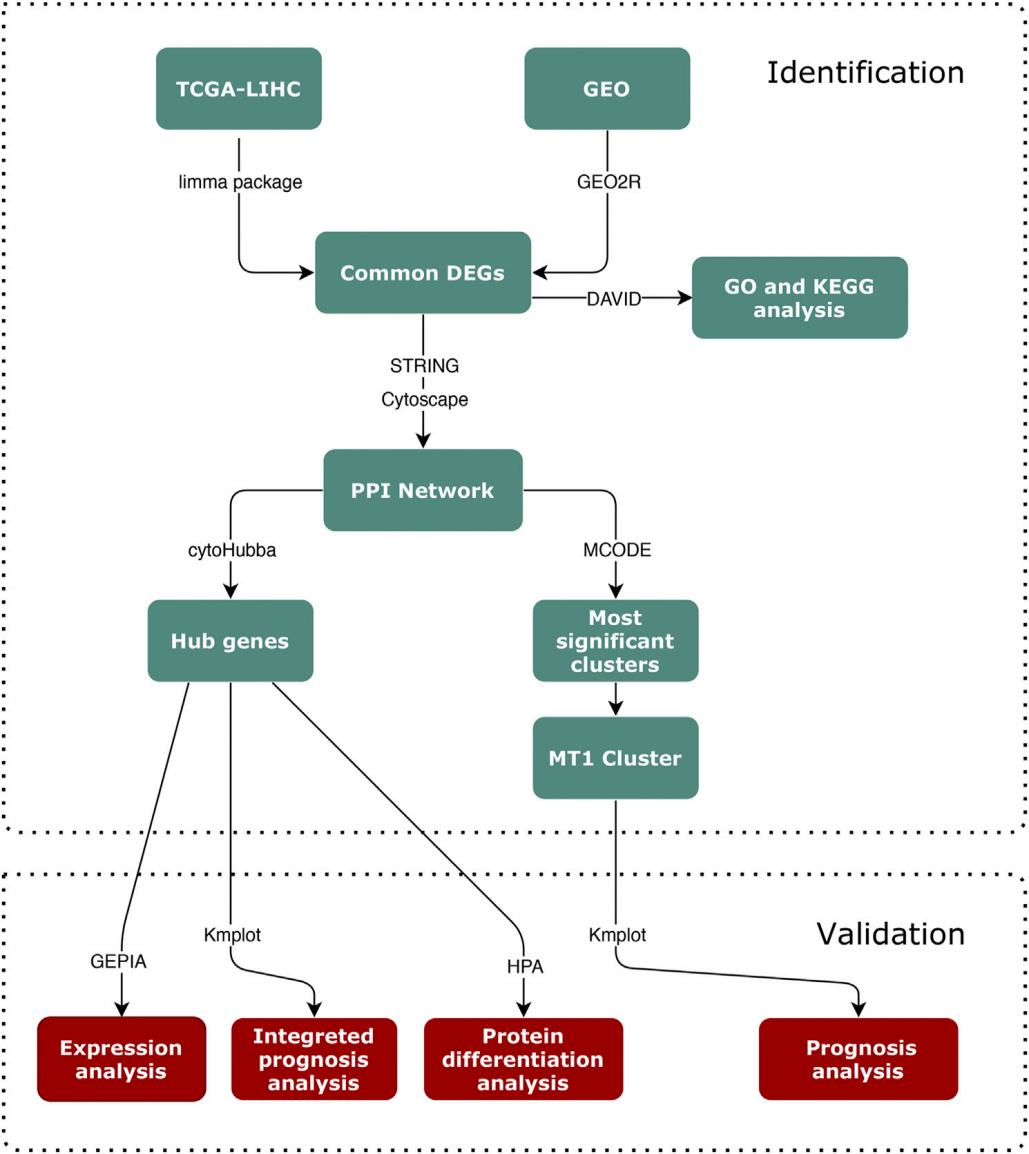
Flowchart of the identification and validation process.

Hence, in our present study, we performed an aCGH analysis to obtain the CNV data of Guangxi HCC patients, and conducted the bioinformatic analysis on HCC datasets from the online database (Figure 1), The Cancer Genome Atlas (TCGA) and Gene Expression Omnibus (GEO). Four MT1s and five hub genes were screened to investigate the underlying connection of MT1 CNV and HCC. Then we investigated the prognostic significance of these genes for HCC outcomes. MT1X, TTK, BUB1, CYP3A4, NR1I2, and CYP8B1 were associated with the prognostic of HCC. Furthermore, the prognostic analysis of these genes in MT1-deletion and non-deletion cases were performed and TTK, BUB1, NR1I2, and CYP8B1 may be the potential biomarkers for HCC with MT1-deletion.

## Methods and Materials

### Patient and Sample Collection

We selected 79 HCC patients (termed Guangxi HCC cohort) with pathologic diagnosis and clinical follow-up results in the Affiliated Tumor Hospital of Guangxi Medical University from 2014 to 2016. As of June 30, 2018, 31 of the 79 patients were alive, 38 died and 10 lost to follow-up. Formalin-fixed paraffin embedded (FFPE) tumor tissue blocks from these patients were collected from the tumor biobank of Guangxi Medical University. This research project was approved by the Medical Ethics Committee of Guangxi Medical University (20170228-31) and research applications of residual specimens followed university policies and laboratory standards [9].

### Array-Based Comparative Genomic Hybridization Analysis

Genomic DNAs of Guangxi HCC cohort were extracted from fresh tissue using Puregene Kit following manufacturer’s instruction (Qiagen Inc., Valencia, CA). DNA concentration was measured using a NanoDrop spectrophotometer (ND-1000, Thermo Fisher Scientific Inc., Waltham, MA) and high molecular weight DNA quality was verified by agarose gel electrophoresis. For each aCGH analysis, 2.5 μg of test genomic DNA from the patient and 2.5 μg of control DNA from a sex-matched or -mismatched healthy individual were used following the manufacturer’s protocol for the Agilent Human Genome aCGH microarray 60 K (Agilent Technologies Inc., Santa Clara, CA). The differential labeling of test and control DNAs, comparative hybridization onto 8 × 60 K Agilent slides, posthybridization wash, slide scanning, image feature extraction were processed following manufacturer’s instruction. The data were analyzed using Agilent CytoGenomics (version 5.0.2.5) with the built-in ADM-2 algorithm set at threshold value of 6, a cut off value of 0.2, and a filter of ten probes. All CNVs except the recognized copy number variants from the HCC cohort of Genomic Variants (http://projects.tcag.ca/variation/) were recorded. The base pair designations from the Agilent 60 K array are according to the Feb. 2009 (GRCh37/hg19) on the UCSC Human Genome browser (http://genome.ucsc.edu/).

### Online Data Collection and Processing

The CNV data of MT1 were from Copy Number Variation category of LIHC cohort from The Cancer Genome Atlas (TCGA, https://www.cancer.gov/tcga) database (termed TCGA-LIHC). The microarray data of GSE112790 and GSE101685 of HCC including tumor and normal samples were from the Gene Expression Omnibus (GEO, https://www.ncbi.nlm.nih.gov/geo/), the online data used in the differential expression gene analysis was shown in Table 1. To avoid errors caused by platform differences, the datasets from GEO were all based on the platform GPL570 ([HG-U133_ Plus_2] Affymetrix Human Genome U133 Plus 2.0 Array).

**TABLE 1:** Online data used in the differential expression gene analysis.

Accession	Platform	Tumor	Normal	Type
TCGA-LIHC	Illumina HiSeq	372		HCC
GSE112790	GPL570	183	15	HCC
GSE101685	GPL570	24	8	HCC

### DEGs Identification and Venn Screening

Limma package is a R package which is widely used in biology field [10]. We used Limma to determine the differentially expressed genes (DEGs) between MT1-deletion and non-deletion samples. |logFC| > 1 and FDR < 0.05 were considered statistically significant. Next, we performed the GEO2R, an official analysis tool to screen DEGs between tumor samples and normal samples of GEO datasets with |logFC| > 1 and adjust *p* Value < 0.05. The common genes among all the three datasets were investigated by VennDiagram package in R [11].

### Protein-Protein Interaction Network Construction and Hub Gene Recognition

We used the STRING (https://string-db.org) database to analyze the up-regulated and down-regulated DEGs and analyzed the protein-protein interaction (PPI) [12]. Cytoscape is a software for visualizing interaction networks and biological pathways [13]. The MCODE plugin was used to find clusters in PPI networks with the degree cutoff, node score cutoff, k-core and max depth as 2, 0.2, 0.2, and 100 as threshold. Moreover, the cytoHubba plugin was used to identify hub genes of the network we imported by calculating the node scores [14]. To get a more reliable result, we analyzed the top 10 nodes with highest degree with all the 12 means. Then we ordered the number of occurrences of these genes, and the genes with the highest occurrence were the most significant hub genes.

### GO and KEGG Pathway Analysis

The Gene Ontology (GO, http://www.geneontology.org/) knowledgebase is a source of information on the functions of genes. Kyoto Encyclopedia of Genes and Genomes (KEGG) is a database resource for understanding high-level functions and utilities of the biological system. To investigate the potential function among the DEGs, we applied GO and KEGG pathway enrichment on the Database for Annotation, Visualization and Integrated Discovery (DAVID, https://david.ncifcrf.gov/), a bioinformatic website that is designed to investigate the biological functions of a number of genes [15].

### Survival and Expression Analysis of Hub Genes

Gene Expression Profiling Interactive Analysis (GEPIA, http://gepia.cancer-pku.cn/) is a web server for cancer and normal gene expression profiling and interactive analysis [16]. The clinical prognosis and expression of the hub genes we found were analyzed by GEPIA and Kaplan-Meier Plotter database (http://kmplot.com) based on TCGA-LIHC data. The prognostic effect of the hub genes in MT1-deletion and non-deletion cases were performed by Survival package in R. Then the Human Protein Atlas (HPA, https://www.proteinatlas.org), a database aims to map all the human proteins in cells, tissues and organs using integration of various omics technologies, were used to analyze the protein expression differentiation between tumor and normal tissue [17].

## Results

### MT1 Deletion Has a High Incidence and Causes Poor Prognosis in Guangxi HCC Cohort

**FIGURE 2:**
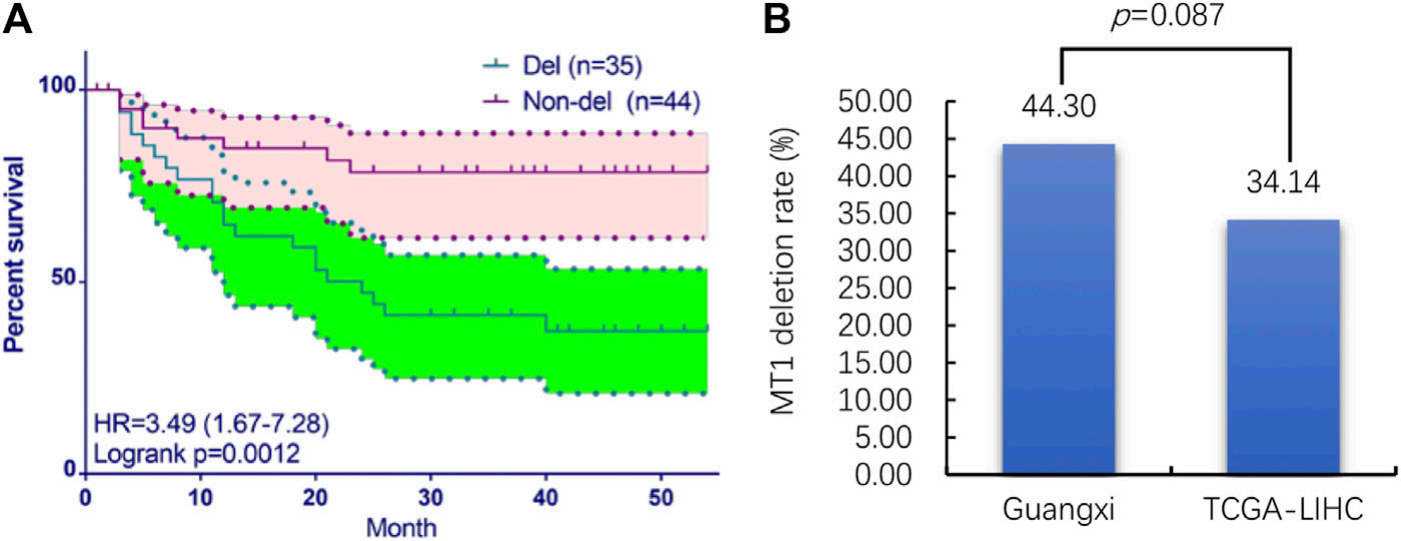
The CNV and prognostic analysis of MT1 deletion in Guangxi HCC cohort and TCGA-LIHC dataset. **(A)** prognostic analysis of MT1 deletion in Guangxi HCC cohort; **(B)** MT1 deletion rate comparing between Guangxi HCC cohort and TCGA-LIHC dataset.

The aCGH analysis results show that MT1 deletion occurred in 35 of the 79 (44.3%) samples. The clinical information of 79 Guangxi HCC patients are displayed in Table 2. Then the prognostic analysis shows that the deletion of MT1 had a significant association with poor prognosis of HCC patients (Figure 2A). Then we performed univariate and multivariate Cox regression analysis in Guangxi cohort (Table 3). The results show that CNV (MT1 deletion), BCLC (Barcelona Clinic Liver Cancer staging) and postoperative recurrence can be independent prognostic factors in HCC. We used the MT1 deletion segment less than −0.2 as the screening threshold, and screened a total of 372 cancer tissue samples in TCGA-LIHC dataset, of which 127 were with MT1 deletion (34.14%). We further compared the MT1 deletion rate of cancer samples from Guangxi HCC cohort and TCGA-LIHC dataset, and found no significant difference between the two rates (*p* > 0.5, Figure 2B). Given the same MT1 deletion rate, we analyzed transcriptome data and clinical follow-up data from TCGA and GEO datasets to identify underlying molecules that may function in MT1 deletion in HCC.

**TABLE 2:** The clinical information of Guangxi cohort.

Characteristic	Frequency/mean ± SD
CNV^a^	
MT1 non-deletion	55.7%
MT1 deletion	44.3%
Age	48.29 ± 11.27
Gender	
Male	91.1%
Female	8.9%
AFP	740.83 ± 649.04
Ki-67	0.36 ± 0.28
Edmenson grade	
II	44.3%
III	55.7%
BCLC	
A	40.5%
B	15.2%
C	44.3%
Lymphatic metastasis	
0-No	96.2%
1-Yes	3.8%
Postoperative recurrence	
0-No	63.3%
1-Yes	36.7%

^a^ AFP, alpha fetoprotein; BCLC, barcelona clinic liver cancer staging; CNV, copy number variation.

**TABLE 3:** The univariate and multivariate Cox regression analysis in Guangxi cohort.

	Univariate analysis		Multivariate analysis	
Variables	HR (95% CI)	*p* value	HR (95% CI)	*p* value
CNV^a^				
MT1 non-deletion	References			
MT1 deletion	2.25 (1.09–4.63)	**0.029**	2.53 (1.17–5.50)	**0.019**
Age	0.979 (0.950–1.010)	0.178		
Gender				
Male	References			
Female	0.918 (0.279–3.02)	0.888		
AFP	1 (0.999–1.00)	0.821		
Ki-67	0.639 (0.162–2.51)	0.521		
Edmenson grade				
II	References			
III	0.691 (0.340–1.40)	0.305		
BCLC				
A	References			
B/C	2.30 (1.46–3.61)	**<0.001**	2.70 (1.60–4.55)	**<0.001**
Lymphatic metastasis				
No	References			
Yes	5.40 (1.59–18.4)	**0.007**	0.875 (0.241–3.18)	0.84
Postoperative recurrence				
No	References			
Yes	3.66 (1.76–7.63)	**<0.001**	6.21 (2.71–14.3)	**<0.001**

^a^ AFP, Alpha fetoprotein; BCLC, barcelona clinic liver cancer staging; CNV, copy number variation; Bold‐italic entries, indicates that the difference is statistically significant.

### 147 Common DEGs Were Screened Through the Intersection of Three Datasets

**FIGURE 3:**
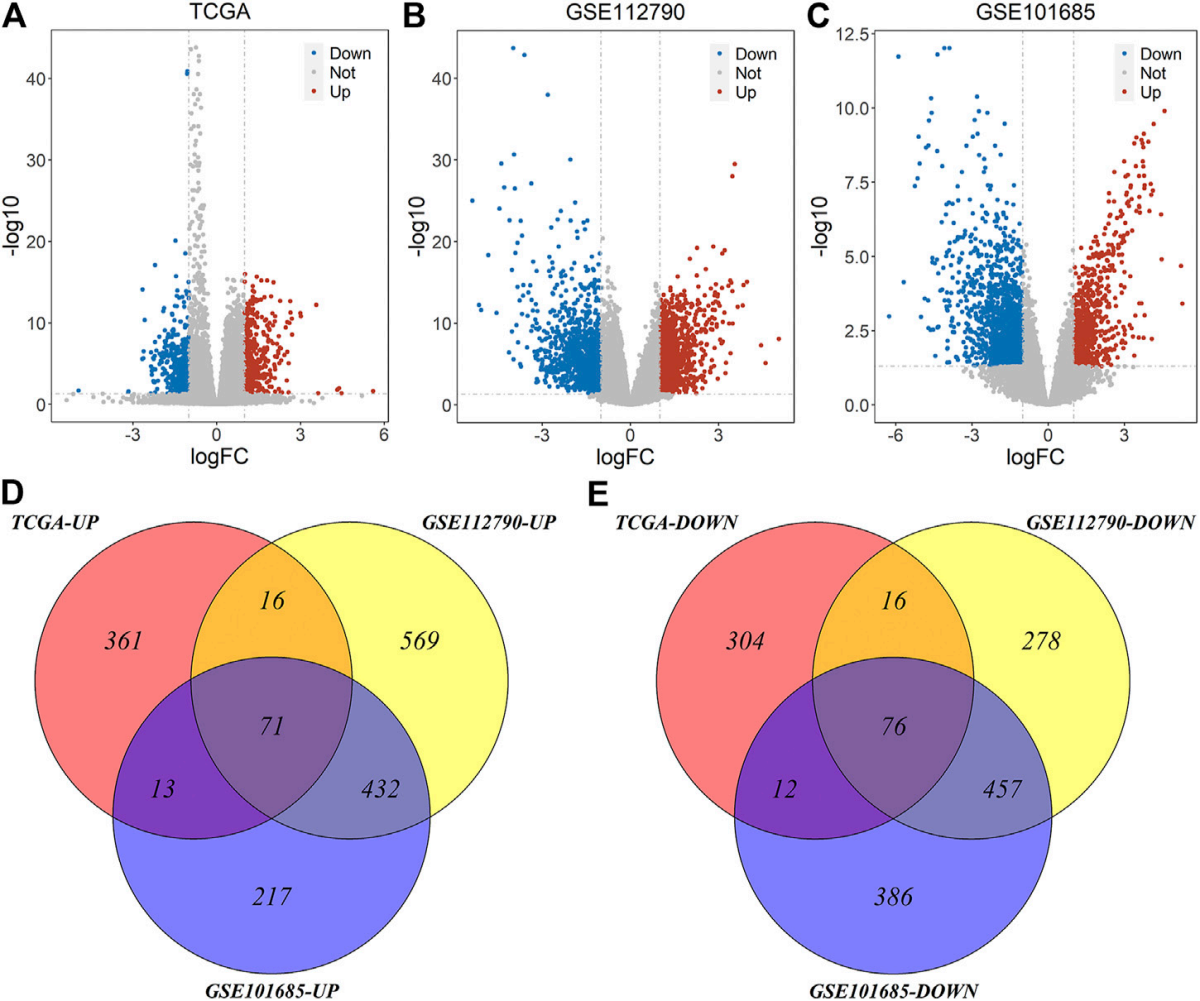
Volcano plot and Venn plot of differentially expressed genes of three datasets. **(A)** Differentially expressed genes of MT1-deletion and non-deletion HCC samples of TCGA-LIHC; **(B–C)** Differentially expressed genes of tumor and normal samples of GSE112790 and GSE101685 **(D–E)** Common differentially expressed genes of three datasets.

Based on the deletion or non-deletion of MT1, we got 869 DEGs from TCGA-LIHC datasets, including 461 up-regulated genes and 408 down-regulated genes. Based on the tumor and normal samples of GEO datasets, we got 1,915 and 1,664 differentially expressed genes in GSE112790 and GSE101685, respectively, of which 1,088 were up-regulated and 827 were down-regulated in GSE112790 and 733 were up-regulated and 931 were down-regulated in GSE101685 (Figures 3A–C). Taking the intersection of these datasets, we got 147 common genes, of which 71 were up-regulated and 76 were down-regulated (Figures 3D,E).

### The Up-, and Down-Regulated DEGs May Be Involved in Cell Division and Metabolism Respectively

**FIGURE 4:**
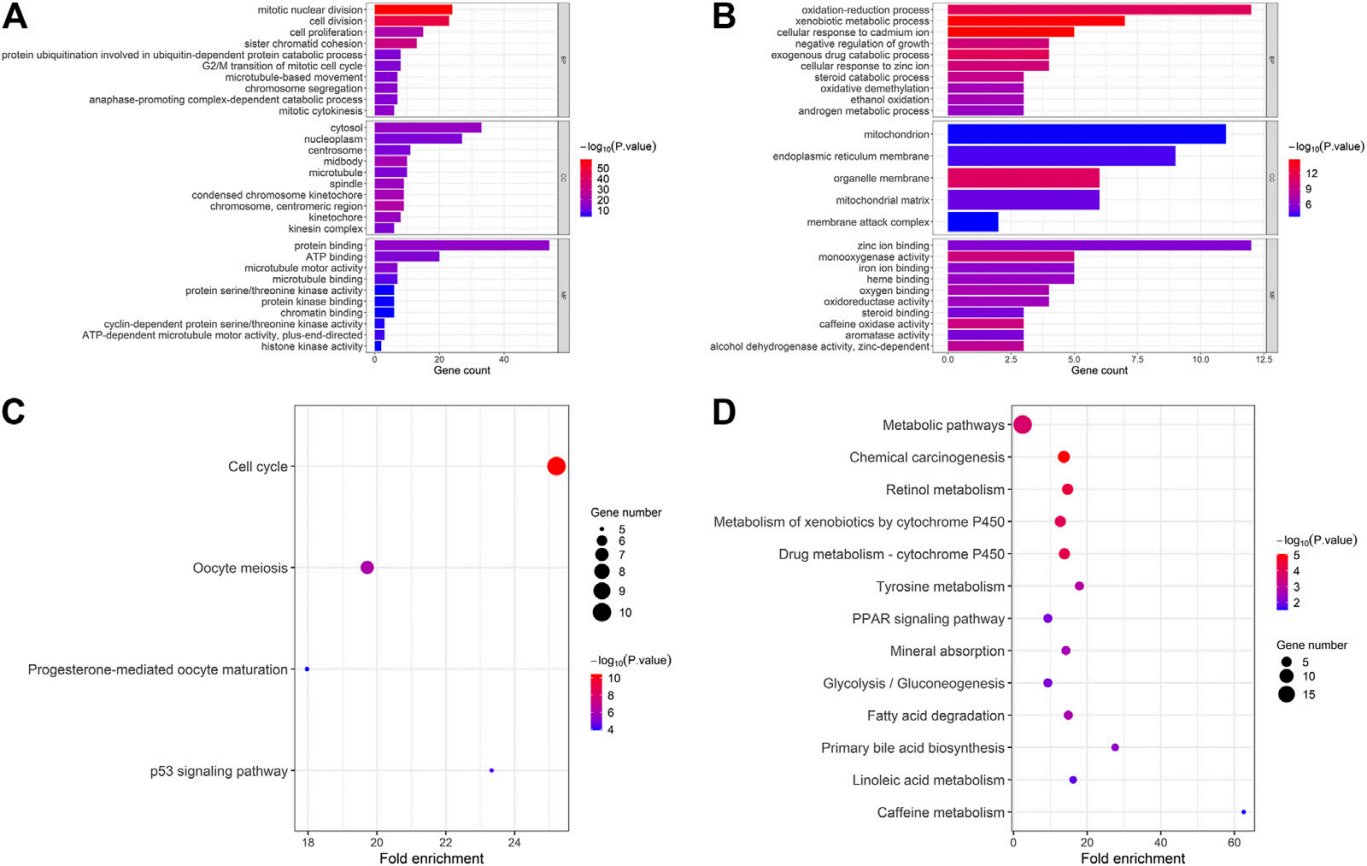
Functional annotation and pathway analysis of common DEGs. **(A)** GO and KEGG pathway enrichment of up-regulated common genes; **(B)** GO and KEGG pathway enrichment of down-regulated common genes. CYP3A4, NR1I2, CYP8B1, TTK, BUB1, and four MT1s were selected as high score hub genes from the 147 common DEGs.

The 147 DEGs we identified were analyzed by DAVID further. As the figure shows, in up-regulated common genes, the major biological processes were “mitotic nuclear division” and “cell division,” the major cellular component were “cytosol” and “nucleoplasm” and the molecular function analysis indicated that these genes were mainly enriched in “protein binding” and “ATP binding” (Figure 4A). The KEGG pathway analysis showed these genes were mostly enriched in cell cycle pathway (Figure 4A). Likewise, in down-regulated common genes, biological process analysis indicated that these genes were mainly associated with “oxidation-reduction process” and “xenobiotic metabolic process,” the major cellular component was “mitochondrion” and the major molecular function was “zinc ion binding” (Figure 4B). Lastly, the KEGG analysis demonstrated that these genes were most significantly enriched in metabolic pathways (Figure 4B).

**FIGURE 5:**
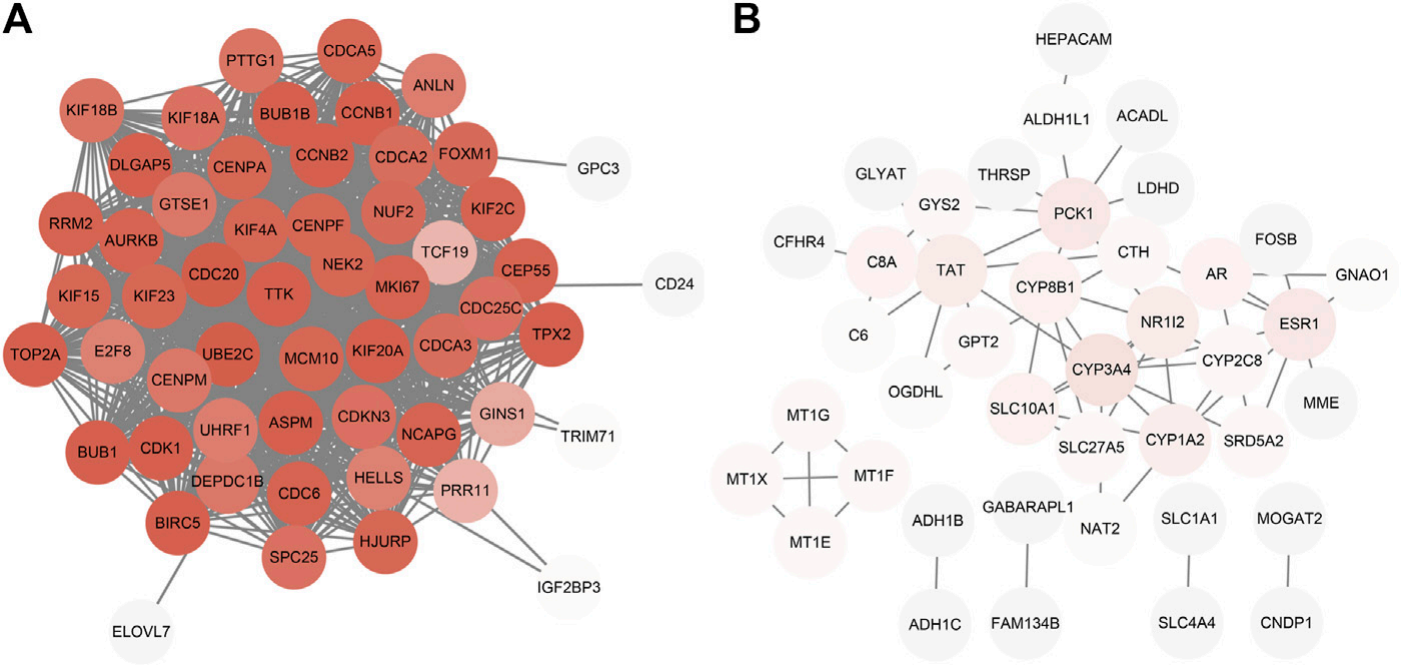
PPI network construction. **(A)** Up-regulated genes; **(B)** Down-regulated genes.

**FIGURE 6:**
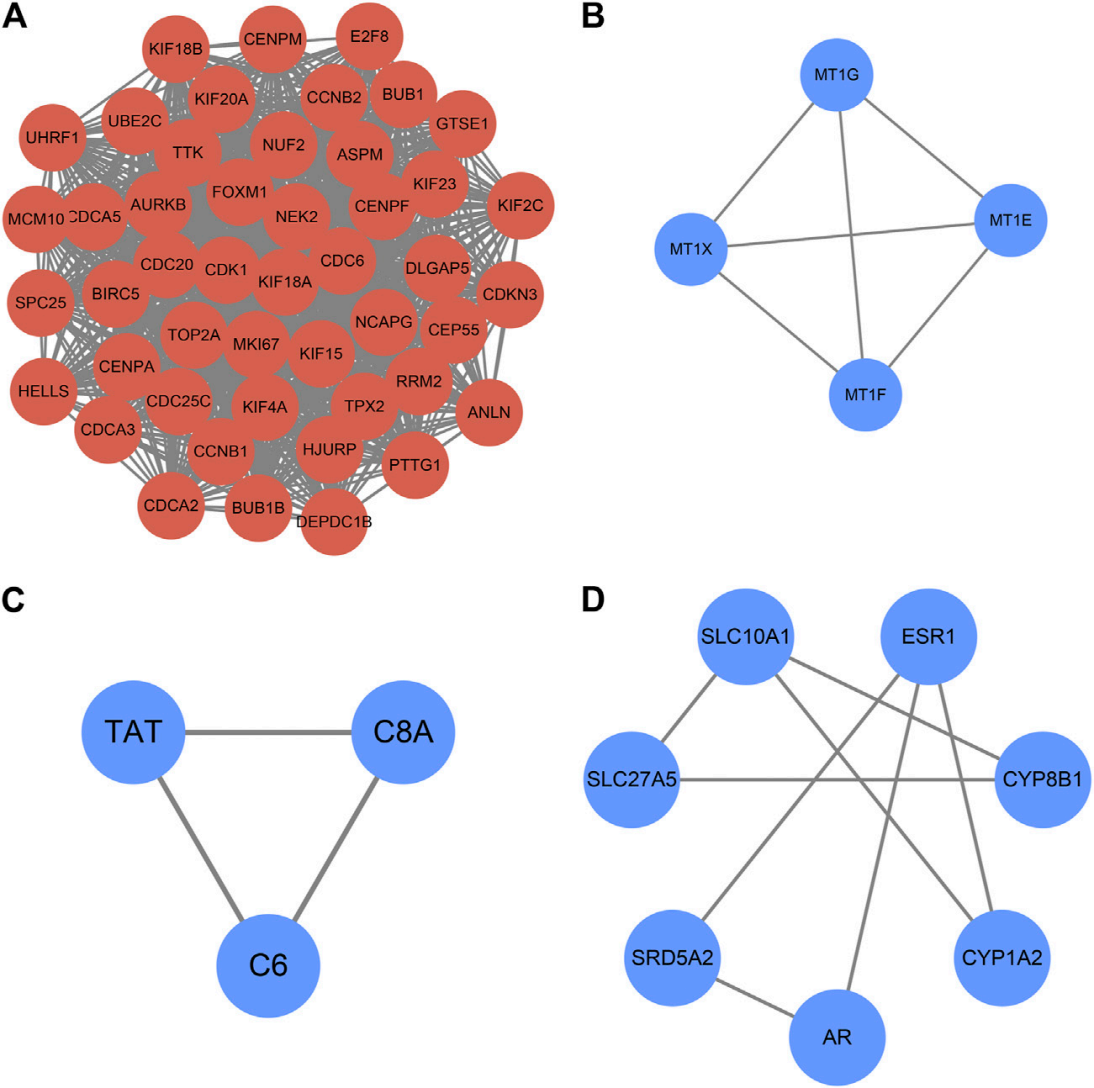
Clusters of MCODE (*K-score >* 2). **(A)** Cluster of up-regulated genes **(B–D)** Clusters of down-regulated genes. The expression and prognostic effect of MT1X and other five hub genes are validated.

The PPI network constructed by Cytoscape is shown in Figure 5. The MCODE plugin was applied to detect the cluster that has the highest score among the PPI network (Figures 6A–D). Finally, we got a cluster with 47 nodes and 1,047 edges in up-regulated genes and three clusters with 4 nodes 6 edges, 3 nodes 3 edges and 7 nodes 8 edges in down-regulated genes, respectively. The most significant cluster with the highest K-core contains four genes including MT1G, MT1E, MT1F, and MT1X, that were down-regulated and closely related to this study. Then the 10 most significant up-regulated hub genes that cytoHubba obtained were, TTK, BUB1, TOP2A, TPX2, DLGAP5, ASPM, UBE2C, CDC20, MKI67, BUB1B, of which TTK and BUB1 ranked first count 10 times were selected as up-regulated hub genes. Likewise, the 10 most significant down-regulated hub genes were CYP3A4, NR1I2, CYP8B1, CYP1A2, ESR1, TAT, PCK1, SLC10A1, AR, CTH, of which CYP3A4, NR1I2, and CYP8B1 ranked first were selected as down-regulated hub genes (Table 4).

**TABLE 4:** Hub genes analysis of common DEGs by Cytoscape, MCODE and cytoHubba.

Regulation	Gene symbol	Counts	Rank	Description	Location
Up	TTK	10	1	TTK protein kinase	6q14.1
	BUB1	10	1	Budding uninhibited by benzimidazoles 1	2q13
	TOP2A	8	3	DNA topoisomerase II alpha	17q21.2
	TPX2	8	3	TPX2 microtubule nucleation factor	20q11.21
	DLGAP5	8	3	DLG associated protein 5	14q22.3
	ASPM	8	3	Abnormal spindle microtubule assembly	1q31.3
	UBE2C	8	3	Ubiquitin conjugating enzyme E2 C	20q13.12
	CDC20	7	8	Cell division cycle 20	1p34.2
	MKI67	5	9	Marker of proliferation Ki-67	10q26.2
	BUB1B	5	9	BUB1 mitotic checkpoint serine/Threonine kinase B	15q15.1
Down	CYP3A4	10	1	Cytochrome P450 family 3 subfamily a member 4	7q22.1
	NR1I2	10	1	Nuclear receptor subfamily 1 group I member 2	3q13.33
	CYP8B1	10	1	Cytochrome P450 family 8 subfamily B member 1	3p22.1
	CYP1A2	9	4	Cytochrome P450 family 1 subfamily a member 2	15q24.1
	ESR1	9	4	Estrogen receptor 1	6q25.1-q25.2
	TAT	9	4	Tyrosine aminotransferase	16q22.2
	PCK1	8	7	Phosphoenolpyruvate carboxykinase 1	20q13.31
	SLC10A1	7	8	Solute carrier family 10 member 1	14q24.1
	AR	6	9	Androgen receptor	Xq12
	CTH	6	9	Cystathionine gamma-lyase	1p31.1

**FIGURE 7:**
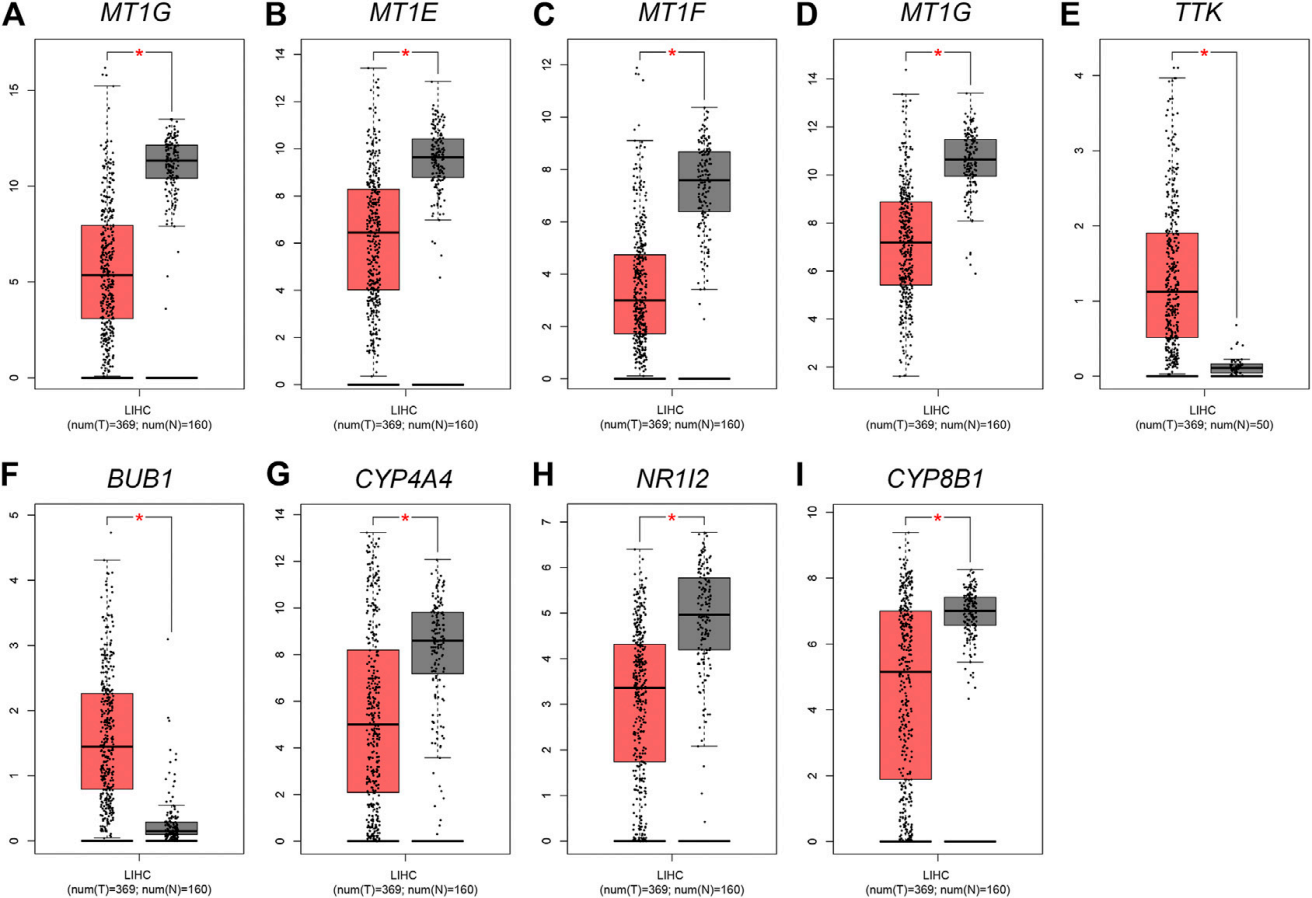
Expression analysis of four MT1s and five hub genes in HCC tissue and adjacent tissue based on TCGA-LIHC data. Orange and gray background represents tumor and normal tissue, respectively; *p < 0.05 **(A)** MT1G; **(B)** MT1E; **(C)** MT1F; **(D)** MT1X; **(E)** TTK; **(F)** BUB1; **(G)** CYP3A4; **(H)** NR1I2; **(I)** CYP8B1.

**FIGURE 8:**
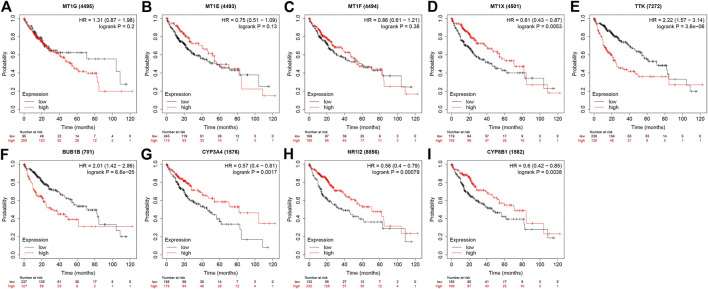
The prognostic effect of four MT1s and five hub genes in Kaplan-Meier Plotter database. **(A)** MT1G; **(B)** MT1E; **(C)** MT1F; **(D)** MT1X; **(E)**TTK; **(F)** BUB1; **(G)** CYP3A4; **(H)** NR1I2; **(I)** CYP8B1.

The expression profile of four MT1s were validated in GEPIA (Figures 7A–D), as well as the prognosis analysis was applied in Kaplan-Meier Plotter (Figures 8A–D). Except for MT1X which was related to the prognosis of HCC, the other three MT1s genes had no association with the prognosis of HCC patients, though all of the four genes were low expressed in HCC.

**FIGURE 9:**
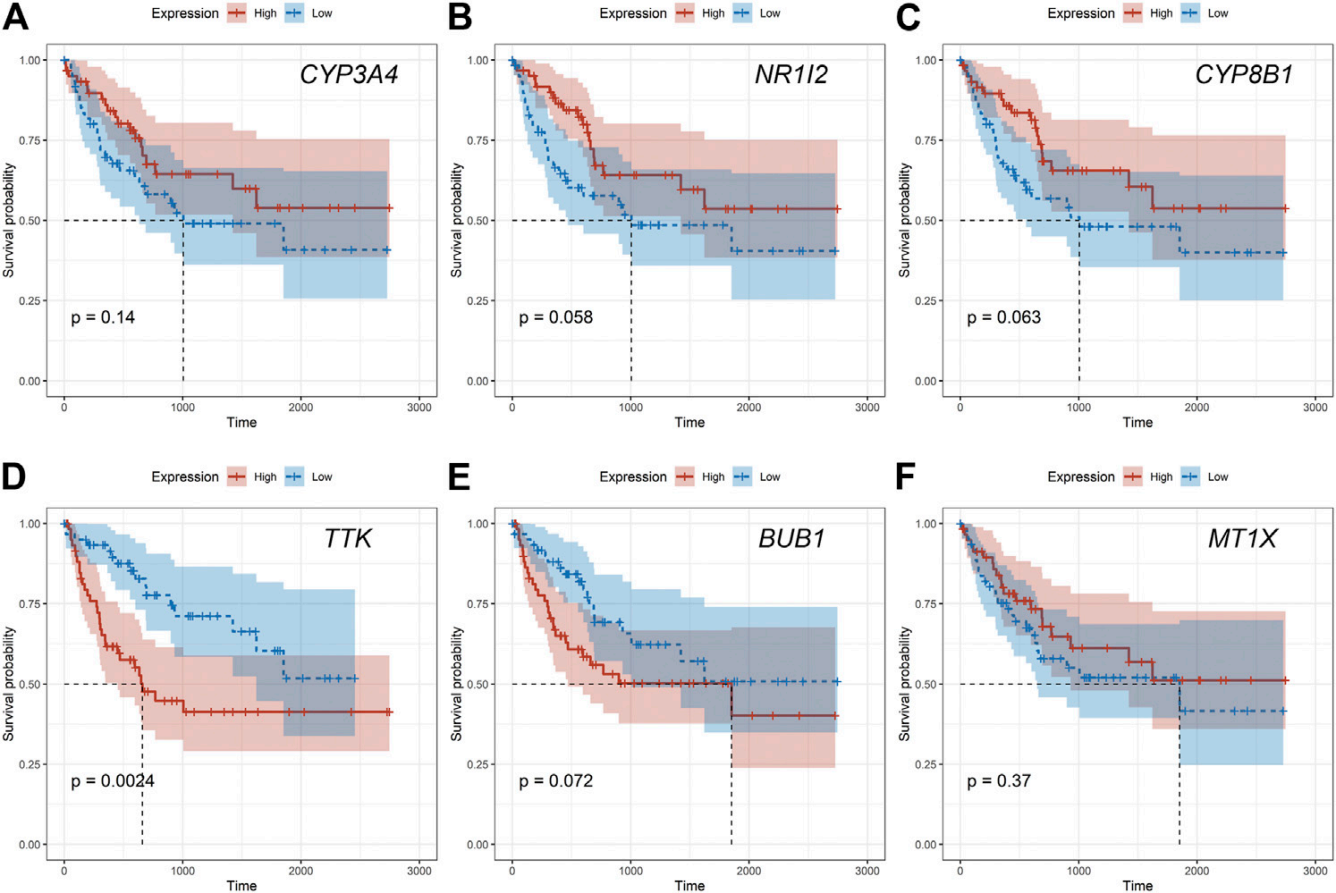
The prognostic effect of hub genes in MT1-deletion cases. **(A)** CYP3A4; **(B)** NR1I2; **(C)** CYP8B1; **(D)** TTK; **(E)** BUB1; **(F)** MT1X.

**FIGURE 10:**
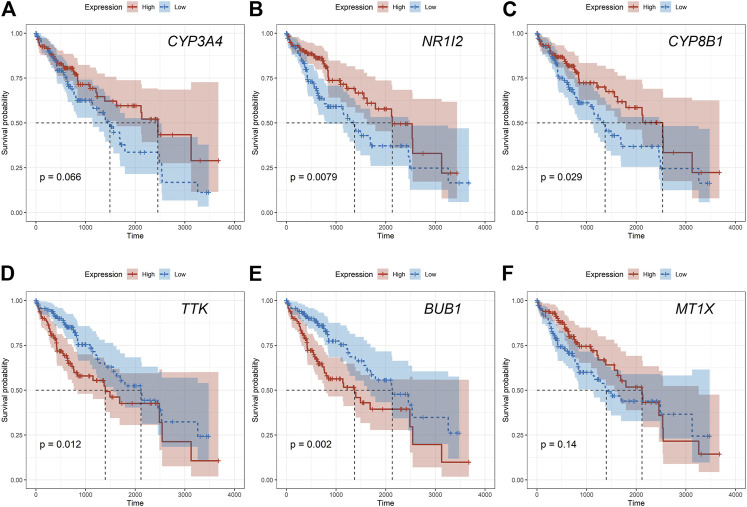
The prognostic effect of hub genes in MT1-non-deletion cases. **(A)** CYP3A4; **(B)** NR1I2; **(C)** CYP8B1; **(D)** TTK; **(E)** BUB1; **(F)** MT1X.

**FIGURE 11:**
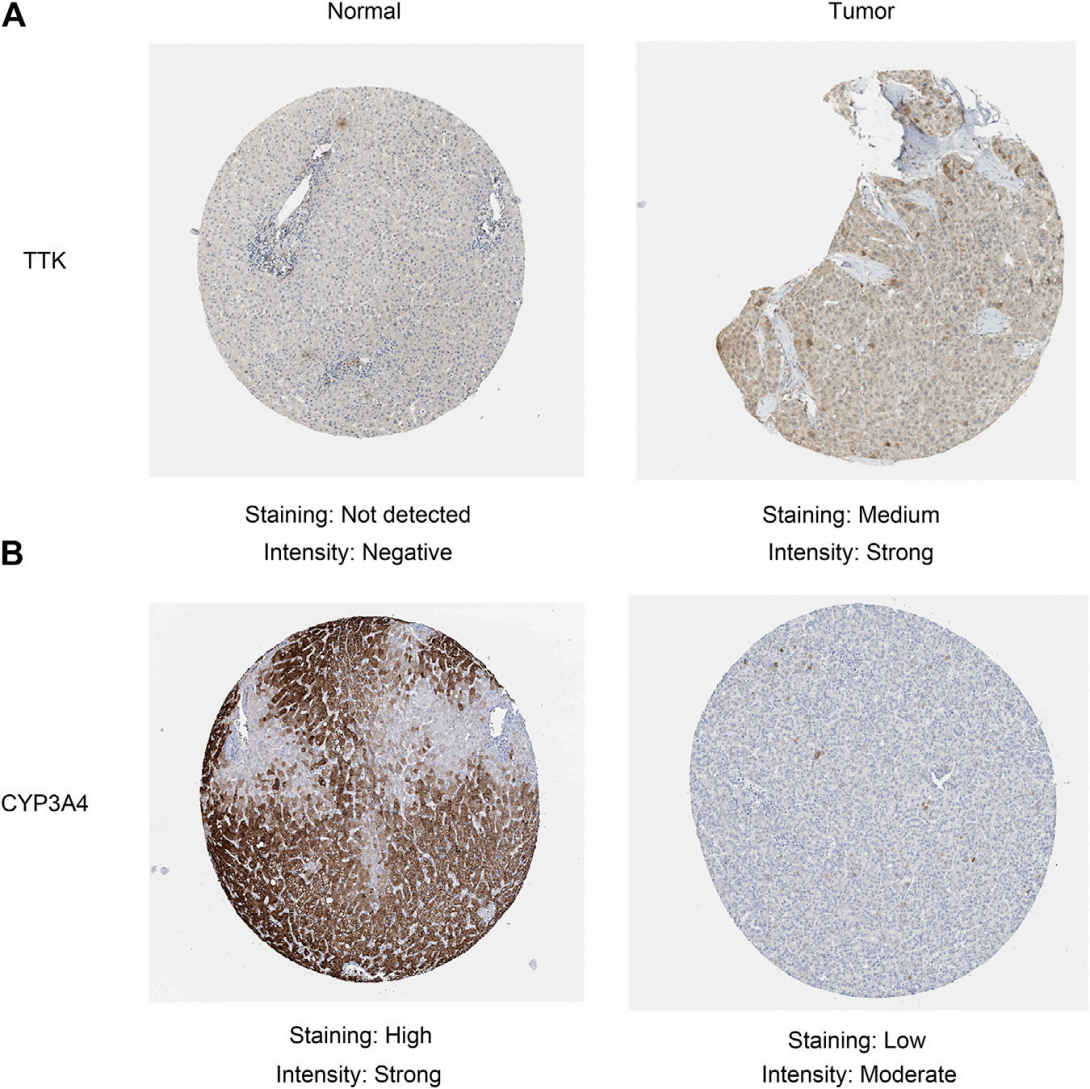
Validation of the protein expression in the Human Protein Atlas database. **(A)** TTK; **(B)** CYP3A4.

Given that only the prognostic effect of MT1X was statistically significant, we explored the expression and prognostic effect of the other five hub genes. The results show that the expressions of two up-regulated hub genes (TTK, BUB1) and three down-regulated hub genes (CYP3A4, NR1I2, CYP8B1) in tumor and normal samples were consistent with their differential expression analysis: TTK and BUB1 were overexpressed, while CYP3A4, NR1I2, and CYP8B1 were low in expression in HCC respectively (Figures 7E–I). Similarly, both up-regulated genes were significantly associated with HCC patients’ prognosis, with higher expression and worse prognosis in HCC (*p < 0.05*) while all the three down-regulated genes were associated with significant worse outcomes (*p < 0.05*) (Figures 8E–I). In order to explore the prognostic effect of these genes in the MT1 deletion and non-deletion groups, we conducted survival analysis in these two groups separately. In MT1-deletion cases, only TTK is statistically significant; in MT1-non-deletion cases, NR1I2, CYP8B1, TTK, and BUB1 are statistically significant (Figures 9, 10). However, the *p* Values of NR1I2, CYP8B1, and BUB1 are all close to 0.05 in MT1-deletion cases. We think that the reason for this result is the MT1-deletion group has fewer samples than the non-deletion group (127 cases compared to 245 cases). At last, we checked the protein expression distribution of these genes in The Human Protein Atlas, TTK and CYP3A4 were found to be differentially expressed in tumor and normal tissues, which TTK was overexpressed in HCC and the expression of CYP3A4 was found decreased in HCC (Figure 11).

## Discussion

HCC is one of the most harmful diseases to human life throughout the world and its pathogenesis is extremely complicated. Multiple biological processes including genetic and environment factors are involved in the development of HCC. Thus, discovering potential biomarkers and molecular mechanisms of HCC genesis and development is vital for diagnosis and treatment. In recent years, as researchers have deepened their understanding of CNV, the application of CNV in HCC research has also increased. However, so far, there is no research on the relationship between the metallothionein family CNV and HCC.

In this study, we demonstrated that MT1 deletion had a high incidence in Guangxi HCC patients and was associated with poor HCC prognosis. Then we screened 147 common DEGs between MT1 deletion DEGs and HCC DEGs form TCGA and GEO datasets, including 71 up-regulated genes and 76 down-regulated genes. The up-regulated DEGs were mainly enriched in cell cycle and the p53 signaling pathway, while the down-regulated DEGs were mainly involved in several metabolic pathways. We have learnt that cancer is characterized by aberrant cell cycle activity, the aberrant activity of cell cycle protein is a hallmark of human cancer [18, 19]. p53 is well known as a tumor suppressor gene, and its abnormal expression is one of the main causes of cancer [20]. What’s more, the metabolic pathway in HCC is generally abnormal and it is essential to the development and growth of HCC [21].

Interestingly, through PPI analysis, we got a four-gene cluster with MT1G, MT1E, MT1F, and MT1X. It has been reported that the MT1 family are abnormally expressed in a variety of cancers including HCC, which have a diverse expression pattern in different type of cancers [22–24]. In HCC, the expression of most MT1 family are significantly down-regulated, and will affect the occurrence and development of HCC [8]. With regard to the four MT1s we identified, studies have confirmed that the expression of MT1G could serve as tumor suppressor by inhibiting cell proliferation and enhancing apoptosis in HCC [25]; MT1F was proven to inhibit the HCC cell growth [26]; Liu et al. demonstrated that MT1X is probably a prognostic biomarker and reduces the progression and metastasis of HCC [27]. Likewise, we validated that four MT1s were all downregulated in HCC with MT1 deletion. Therefore, the four MT1s (MT1G, MT1E, MT1F and MT1X) are down regulated in tumor samples, at the same time, they are also down regulated in tumor samples with MT1 deletion, indicating that MT1 deletion may affect the expression of its genes in HCC. However, the prognosis results showed that the four MT1s had weak correlations with HCC patient prognosis, except MT1X. It may be due to differences between groups caused by different datasets, it requires further effort to investigate. Afterward, we turned our attention to other common DEGs to explore new biomarkers which possibly function in HCC.

Then five genes form PPI network were selected by comprehensive analysis as hub genes by cytoHubba, including TTK, BUB1, CYP3A4, NR1I2 and CYP8B1. TTK Protein kinase, also known as MPS1 is the core component of the spindle assembly checkpoint, which functions to ensure the chromosomes are properly separated [28]. A number of studies have shown that TTK has an important role in cancer, including HCC [29–32]. Budding uninhibited by benzimidazoles 1 (BUB1) encodes serine/threonine protein kinases that are crucial in spindle assemble checkpoint [33], as well as investigations have demonstrated the role in oncogenesis of BUB1 mutation in several cancers [34]. Whereas, the role of BUB1 in carcinogenesis and development of HCC has not been well studied. In this study, we found BUB1 was significantly up-regulated and associated with poor prognosis in HCC with MT1 deletion, which may be involved in HCC carcinogenetic process.

CYP3A4 and CYP8B1 are the members of cytochrome P450 family which may rely on the function of metabolic carcinogens to play a vital role in chemoprevention, carcinogenesis, cancer therapy and metastasis [35]. CYP3A4 has been well studied for decades. Sandström et al. [36] have reported that the activation of CYP3A4 in colorectal cancer cells may affect tumor sensitivity to certain anti-colon cancer drugs. In breast cancer, Murray and colleagues found high expression of CYP3A4 was associated with a poor prognosis [37]. Nevertheless, Fujimura and colleagues verified that lower immunoreactivity score showed a poorer prognosis in prostate cancer patients [38]. Moreover, CYP3A4 has been elucidated to be low expressed and as a biomarker for predicting poor prognosis in HCC [39, 40]. Nuclear receptor subfamily 1 group I member 2 (NR1I2) is a member of nuclear receptor family that regulates the expression of metabolizing and detoxifying enzymes [41, 42]. It is reported that the expression of NR1I2 was often related to chemoresistance in various cancer cells including HCC [43–46]. In addition, NR1I2 is also the main regulator of CYP3A4 [47]. The researches on CYP8B1 are currently scarce. Some studies demonstrated that CYP8B1 transcription was regulated by bile acids, which participated in lipid metabolisms and other liver diseases [48]. Whereas the role of CYP8B1 in HCC remains uncharted. Our results revealed that CYP8B1 was low in expression and had poor prognostic effect in HCC. Next, we used database analysis to verify the differential expression of these key genes in HCC tissue and adjacent normal tissue. Various results indicated that these genes were significantly differentially expressed in cancer and adjacent tissue, and significantly affected the prognosis of HCC. The above results indicated that MT1 deletion was related to the poor prognosis of HCC, which was possible mainly caused by the regulation of cell proliferation and metabolism through the differential expression of CYP3A4, TTK, NR1I2, CYP8B1, and BUB1.

This study also has some shortcomings, for example, we need to further verify the impact of these hub genes on HCC by experiment, and explore the specific mechanism between MT1 deletion and its expression in more depth.

In summary, this article demonstrated MT1-deletion can be an independent prognostic factor in HCC, screened 147 differential expression genes through aCGH and bioinformatics, and found multiple genes may be related to MT1 deletion in HCC. Here, we bridged the gap between the MT1 deletion and HCC, MT1 deletion may cause abnormal expression of genes (TTK, BUB1, NR1I2, CYP8B1) in HCC, thus affecting the prognosis of MT1 deleted HCC patients.

## Data Availability

Publicly available datasets were analyzed in this study. This data can be found here: https://www.cancer.gov/tcga https://www.ncbi.nlm.nih.gov/geo/.
